# Identification of immunogenic proteins and evaluation of recombinant PDHA1 and GAPDH as potential vaccine candidates against *Streptococcus iniae* infection in flounder (*Paralichthys olivaceus*)

**DOI:** 10.1371/journal.pone.0195450

**Published:** 2018-05-30

**Authors:** Xiuzhen Sheng, Min Liu, Haibo Liu, Xiaoqian Tang, Jing Xing, Wenbin Zhan

**Affiliations:** 1 Laboratory of Pathology and Immunology of Aquatic Animals, KLMME, Ocean University of China, Qingdao, P. R. China; 2 Laboratory for Marine Fisheries Science and Food Production Processes, Qingdao National Laboratory for Marine Science and Technology, Qingdao, P. R. China; Institute of Oceanology, Chinese Academy of Sciences, CHINA

## Abstract

*Streptococcus iniae* is a major Gram-positive pathogen that causes invasive disease in fish worldwide. In this study, in order to identify immunogenic proteins for developing highly effective vaccine against *S*. *iniae*, whole-cell lysate proteins of *S*. *iniae* were analyzed by western blotting using flounder anti-*S*. *iniae* antibodies, and two positive protein bands of molecular weight 37 kDa and 40 kDa were screened, which were identified as pyruvate dehydrogenase E1 subunit alpha (PDHA1), BMP family ABC transporter substrate-binding protein (BMP) and L-lactate dehydrogenase (LDH), as well as ornithine carbamoyltransferase (OCT), lactate oxidas (LOx) and glyceraldehyde-3-phosphate dehydrogenase (GAPDH) by mass spectrometry. Subsequently, the six recombinant proteins were produced and used to immunize healthy flounder, and the relative percent survival (RPS) value was 72.73%, 27.27%, 36.36%, 9.09%, 36.36% and 63.64% respectively after intraperitoneal challenge with live *S*. *iniae*, revealing that rPDHA1 and rGAPDH produced higher relative percent survival than formalin-killed *S*. *iniae* (36.36%). To further investigate the protective efficacy of rPDHA1 and rGAPDH, the proliferation of surface membrane immunoglobulin-positive (sIg^+^) lymphocytes in peripheral blood leucocytes, the total serum IgM, specific IgM against *S*. *iniae* and RPS were detected. The results showed that rPDHA1, rGAPDH and formalin-killed *S*. *iniae* significantly induced the proliferation of sIg^+^ lymphocytes, the production of total serum IgM and specific IgM as compared with the control group, and rGAPDH and rPDHA1 provide higher RPS (62.5% and 75%, respectively) again. These results demonstrated that rPDHA1 and rGAPDH are promising vaccine candidates against *S*. *iniae* infection in flounder.

## Introduction

*Streptococcus iniae* is a Gram-positive pathogen, which infects a wide range of marine and freshwater fish [1], including flounder (*Paralichthys olivaceus*) [2], resulting in serious economic losses around the world [3, 4]. *S*. *iniae* is also known to be an opportunistic human pathogen that can cause soft tissue infections [5, 6]. Nowadays, vaccination is generally considered to be an effective method to control aquatic disease for the advantages of safety, environmental friendliness, and long-term efficacy of protection [7, 8], and frequent outbreaks of streptococcus have highlighted the urgent need to develop a highly protective vaccine. Currently, there are several types of vaccine, including formalin-killed vaccine, modified live vaccine, DNA vaccine and subunit vaccine, are used to control streptococcosis. It has been reported that the fish vaccinated with formalin-killed *S*. *iniae* can produce a higher protection compared with the control group [9, 10]. In addition, the high protective capacity of ΔsimA and ΔPGM mutant as a live attenuated vaccine candidate against *S*. *iniae* are demonstrated in aquaculture [11, 12]. These two kinds of vaccines have been developed as commercial vaccines for prevention of Streptococcus disease [13]. Moreover, DNA vaccine is well-known for its advantages of inducing humoral and cellular immune responses, requiring no adjuvants and providing longer protection [14, 15], and a few proteins have been constructed as DNA vaccine against *S*. *iniae*, such as Sia10 and enolase, which could provide high protective efficacy to fish [16, 17]. Additionally, subunit vaccines against *S*. *iniae* are studied due to its safety and less side effects [18]. Several proteins of *S*. *iniae*, including glyceraldehyde-3-phosphate dehydrogenase (GAPDH), Sip 11, MtsB, C5a peptidase (ScpI), enolase (ENO) and fructose-bisphosphate aldolase (FBA), have been confirmed to be immunogenic [19–22], among which some recombinant proteins can provide effective protection in laboratory trials and may be useful as vaccine candidates against *S*. *iniae* infection [23, 24]. Though several effective subunit vaccine candidates have been obtained, no commercial subunit vaccine is available. Therefore, it is important to identify immunogenic proteins to develop highly protective vaccines against *S*. *iniae*.

Recently, several immunogenic proteins of *Edwardsiella tarda* [25, 26], *Vibrio anguillarum* [27, 28] and *V*. *ichthyoenteri* [29] that could provide high immunoprotection as potential vaccine antigens have been reported in flounder model in our laboratory. As one part of these studies on vaccine candidates against different bacterial pathogens of flounder, the present study aimed to identify and obtain protective antigens as effective vaccine candidates against *S*. *iniae*. Considering the immune responses and antibody isotypes between fish and mammals might be different, as described previously that some antigenic proteins identified by rabbit polyclonal antiserum failed to react with fish antiserum [30], the immunogenic proteins were identified with immunoproteomic approach using flounder anti-*S*. *iniae* antibodies to analyze the whole-cell lysate proteins of *S*. *iniae* by western blotting, and then the screened immunogenic proteins were analyzed by mass spectrometry and expressed in *Escherichia coli* BL21 (DE3). The recombinant immunogenic proteins with a higher relative percent survival (RPS) than formalin-killed cells (FKC) of *S*. *iniae* were preliminarily selected after challenge infection with live *S*. *iniae* in immunized flounder. After that, the immune responses of flounder vaccinated with the selected recombinant proteins, including the proliferation of sIg^+^ lymphocytes and the production of total and specific IgM were further evaluated.

## Materials and methods

### Ethics statement

The usage of fish was in strict accordance with the recommendations of the Guidelines for the Use of Experimental Animals of Ocean University of China. The protocol for animal care and handling used in this study was approved by the Committee on the Ethics of Animal Experiments of Ocean University of China (Permit Number: 20141201). Before sacrificing and handling, experimental fish were anesthetized with 100 ng/ml ethyl 3-aminobenzoate methanesulfonic acid (MS222, Sigma, USA), and all efforts were made to minimize suffering. The NC3Rs ARRIVE Guidelines checklist is presented in the S1 Checklist.

### Fish

Healthy flounder (25 ± 5 g), confirmed no *S*. *iniae* infection by standard microscopical and bacteriological examination before, were obtained from a fish farm in Rizhao, Shandong province of China, there, the fish were cultured in relatively strict conditions and the majority of fish had similar size and weight. The fish were maintained in tanks with continuous aerated and running seawater at 21 ± 1 ^o^C for one week before experiment.

Healthy flounder (750 ± 50 g) were purchased and cultured as described above, and used to produce flounder anti- *S*. *iniae* antibodies after acclimated to the laboratory environment for one week.

### Bacterial culture and bacterin preparation

The pathogenic strain of *S*. *iniae* was stored in saline with 15% glycerol at -80 ^o^C in our laboratory. The strain was cultured in brain-heart infusion (BHI) medium at 28 ^o^C for 24 h. The bacterial suspension was obtained by centrifugation at 8000 ×*g* for three times at 4 ^o^C and washing, and then resuspended with phosphate-buffered saline (PBS, pH = 7.2). The formalin killed cells of *S*. *iniae* were prepared as described previously [31]. Briefly, the bacterial suspension was treated with 0.5% formalin (v/v) for 72 h at 4 ^o^C, and the inactivated bacteria was confirmed by incubating the solution on BHI agar at 28 ^o^C. The FKC were obtained by centrifuging at 8000 ×*g* for 15 min at 4 ^o^C and washing three times with PBS, and then the concentration of FKC was adjusted to 1.0 × 10^10^ cfu/ml and stored at -80 ^o^C until use. The safety of FKC was checked by intraperitoneal injection into healthy flounder at 0.2 ml/fish (1.0 × 10^8^ cfu/ml), and no adverse reactions were found within 20 days. Live *S*. *iniae* of 1.0 × 10^7^ cfu/ml was determined by investigating the lethal dose (LD50) in flounder, which was investigated by our previous work [32].

### Antibodies

To obtain flounder anti-*S*. *iniae* antibodies, the FKC (1.0 × 10^8^ cfu/ml) was mixed with an equal volume of Freund’s complete adjuvant (FCA), then injected into the healthy flounder. The boost immunization was performed at week 2 after the initial immunization. At week 4 after vaccination, blood was collected from the executed flounder and placed at 4 ^o^C overnight, and then the serum was obtained by centrifugation at 5000 ×*g* for 20 min at 4 ^o^C and stored at -20 ^o^C for later use.

Mouse monoclonal antibodies (mAb) against flounder serum IgM 2D8 were previously produced in our laboratory [33] and used in this experiment.

### Western blotting and mass spectrometry analysis

Whole-cell lysate proteins of *S*. *iniae* (1.0 × 10^10^ cfu/ml) were subjected to sodium dodecyl sulfate-polyacrylamide gel electrophoresis (SDS-PAGE), one was stained with Coomassie blue, and the other one was transferred onto a polyvinyldifluoride (PVDF) membrane. The membrane was blocked with PBS containing 4% bovine serum albumin (BSA) for 2 h at 37 ^o^C and washed three times with PBS containing 0.05% Tween-20 (PBST), and then incubated with flounder anti-*S*. *iniae* serum or healthy flounder serum as the control for 1 h at 37 ^o^C. After three washes with PBST, the membrane was incubated with mouse anti-serum IgM mAb 2D8 for 1 h at 37 ^o^C, and then washed three times as above. Subsequently, the membrane was incubated with goat-anti-mouse Ig-alkaline phosphatase (Merck Millipore, Darmstadt, Germany) diluted 1:5000 in PBS for 1 h at 37 ^o^C and washed again. Finally, the bands were visualized with freshly prepared substrate solution (100 mM NaCl, 100 mM Tris and 5 mM MgCl_2_, pH 9.5) containing nitroblue tetrazolium (NBT, Sigma, St. Louis, MO, USA) and 5-bromo-4-chloro-3-indolyphosphate (BCIP, Sigma, St. Louis, MO, USA) for 5 min and terminated by washing with distilled water.

According to the positive band in the PVDF membrane, the relevant 37 kDa and 40 kDa band were excised from the gels for protein identification using mass spectrometry by Shanghai Sangon Biotech (China).

### Production and analysis of recombinant proteins

Based on the results of mass spectrometry analysis and amino acid sequence alignment, the specific primers were designed to amplify the open reading frame (ORF) of the six identified proteins excluding the region coding for signal peptide as listed in Table 1. PCR amplification products were purified and ligated to PEASY-E1 vector by pEASY-E1 Expression Kit (Transgen) to construct recombinant plasmids, and then transformed into *E*. *coli* BL21 (DE3). The positive recombinant strain was cultured in LB medium to a mid-logarithmic phase and the expression of recombinant protein was induced by adding 100mM isopropyl-β-D-thiogalactopyranoside (IPTG) for 10 h. His-tagged recombinant proteins were purified using His Trap™ HP Ni-Agarose (GE healthcare China, Beijing, China) according to the manufacturer’s instruction. The purified protein was renatured in TBS glycerol buffer (50 mM Tris-HCl, 50 mM NaCl, 10% glycerol, and 6, 4, 2, 0 M urea, pH 8.0) by four dialysis steps and each dialysis step was performed at 4 ^o^C for at least 12 h. Finally, the proteins were analyzed by SDS-PAGE and visualized with Coomassie brilliant blue R-250 staining [34]. Western blotting as described above was used to analyze the immunogenicity of recombinant proteins.

**Table 1:** Primers used in this study.

Primer NO.	Primer Name	Primer Sequence(5’-3’)	Accession No.
1	pyruvate dehydrogenase E1 subunit alpha -F	ATGGTAACAGTTTCGAAAGAAAAAC	AGM98559.1
2	pyruvate dehydrogenase E1 subunit alpha -R	TTAGTCAACCCAAATATCTTCGTATG	
3	lipoprotein -F	ATGAACAAGAAATTTATTGGTCTCG	AGM98765.1
4	lipoprotein -R	TTATTTTTCAGGAACTTTAATGTCTC	
5	lactate dehydrogenase -F	ATGACTGTAACCAAACAACACA	AGM98841.1
6	lactate dehydrogenase -R	TTAATTTTTAGCAGCAGAAGC	
7	ornithine carbamoyltransferase -F	ATGACTCAAGTATTCCAAGGACGTAG	AGM98316.1
8	ornithine carbamoyltransferase -R	TTATACTTTAGGGATAAAGAGGTTACCT	
9	lactate oxidase -F	ATGGAAAATAAATCAGAAATGATAAAT	AGM99277.1
10	lactate oxidase -R	TTAATCTAATTTTAAAGCATTTTGGC	
11	glyceraldehyde-3-phosphate dehydrogenase -F	ATGGTAGTTAAAGTTGGTATTAACGG	AGM99660.1
12	glyceraldehyde-3-phosphate dehydrogenase -R	TTATTTAGCAATTTTTGCGAAGTAC	

### Fish vaccination and sampling

The fish (25 ± 5 g) were equally divided into eight groups (30 fish/group). For vaccination, the concentrations of purified recombinant proteins were all adjusted to 1mg/ml, and each experimental group was intraperitoneally injected with 100 μl purified recombinant protein (1 mg/ml) or FKC (1.0 × 10^8^ cfu/ml) mixed with equal volume of FCA. The control group was intraperitoneally injected with 100 μl of PBS and FCA (1:1). At day 35 post-vaccination, fish were challenged with 100 μl live *S*. *iniae* (1.0 × 10^7^ cfu/ml) by intraperitoneal injection. Mortalities were monitored over a period of 15 days after the challenge and the relative percent survival (RPS) rates were calculated according to the formula: RPS = (1 - (%mortality in vaccinated fish / % mortality in control fish)) × 100% [35].

To further evaluate the immune response induced by the screened recombinant protein that could elicit higher level of immnoprotection, the flounders (75 fish/group, with three replicates in each group) were vaccinated with FKC, rPDHA1, rGAPDH or PBS as described above. At week 1, 2, 3, 4, 5, 6 and 7 after immunization, six fish in each group was randomly sampled and anaesthetized with MS-222, and then the blood was collected from the caudal vein. For serum isolation, blood was allowed to clot overnight at 4 ^o^C, then the serum was obtained by centrifugation at 5000 ×*g* for 20 min and stored at -20 ^o^C until use for ELISA assay. The PBLs of vaccinated flounder were isolated as described in previous study [26]. Briefly, blood was diluted 1:1 in 65% RPMI-1640 (Gibco) containing 20 IU/ml heparin, 0.1% (w/v) NaN_3_ and 1% (w/v) BSA and stored at 4 ^o^C for 1 h, then centrifuged at 100 ×*g* for 20 min to discard the red cells. Subsequently, the cell suspensions were laid over the Percoll density media between 1.020 g/cm^3^ and 1.070 g/cm^3^. After centrifugation at 840 ×*g* for 30 min, a leukocyte fraction was collected from the 1.020–1.070 g/cm^3^ interface. The leukocyte fraction was washed three times with PBS containing 5% (v/v) Newborn Calf Serum for 5 min at 640 ×*g* to remove Percoll, and then the cells were resuspended in PBS and used for fluorescence-activated cell sorter (FACS) analysis. Moreover, at day 35 post-vaccination, fish were challenged with 100 μl live *S*. *iniae* (1.0 × 10^7^ cfu/ml) by intraperitoneal injection, and mortalities were monitored over a period of 15 days after the challenge and the RPS was calculated again as described above.

### Flow cytometric immunofluorescence analysis

The isolated peripheral blood leucocytes (PBLs) were diluted to 1.0 × 10^7^ cells/ml, then incubated with anti-serum IgM mAb 2D8 (1:1000 diluted in PBS) for 1.5 h at 37 ^o^C. Subsequently, cells were washed three times with PBS containing 5% (v/v) Newborn Calf Serum, and incubated with goat-anti-mouse Ig-FITC (1:256 diluted in PBS, Sigma) for 45 min at 37 ^o^C. After washing as above, the cell suspensions were analyzed with an Accuri C6 cytometer (BD Accuri™, Piscataway, NJ, USA) to determine the percentage of the surface immunologlobulin-positive (sIg+) lymphocytes in PBLs. Myeloma culture supernatant instead of anti-serum IgM mAb 2D8 was used as negative control.

### Detection of total and specific IgM in serum by ELISA

For the determination of total IgM, the serum (1:100 diluted in PBS) sampled from vaccinated fish at different time points were added 100 μl per well and incubated for 1h at 37 ^o^C. Following three washes, 100 μl anti-serum IgM mAb 2D8 (1:1000 diluted in PBS) was added for serum total antibody detection. After incubation at 37 ^o^C for 1 h and washing, 100 μl goat-anti-mouse Ig-alkaline phosphatase conjugate (Sigma) diluted 1:5000 in PBS was added and incubated for 1 h at 37°C. After the last three washes, 100 μl 0.1% (w/v) *p*-nitrophenyl phosphate (*p*NPP, Sigma, USA) in 50 mM carbonate-bicarbonate buffer (pH = 9.8) containing 0.5 mM MgCl_2_ was added to each well and incubated at room temperature for 30 min in the dark. The reaction was stopped by adding 50 μl per well of 2 M NaOH and absorbance was measured with an automatic ELISA reader (TECAN, Männedorf, Switzerland) at 405 nm.

For detecting the specific IgM against *S*. *iniae*, wells of flat-bottom microplates (96-wells, Costar) were coated with 100 μl/well of live *S*. *iniae* (1.0 × 10^9^ cfu/ml) for 3 h at 37 ^o^C. Then the serum of vaccinated fish, anti-serum IgM mAb 2D8 and goat-anti-mouse Ig-alkaline phosphatase conjugate were successively incubated and washed, and the absorbance was measured as described above.

### Statistical analysis

The statistical analysis was performed using Statistical Product and Service Solution (SPSS) software (Version 20.0; SPSS, IBM, Armonk, NY, USA), differences between different groups were analyzed with one-way analysis of variance (ANOVA) and the results were expressed as mean ± SEM. In all cases, the significance level was defined as *p* < 0.05.

## Results

### Immunogenic protein identification of *S*. *iniae*

**Fig 1:**
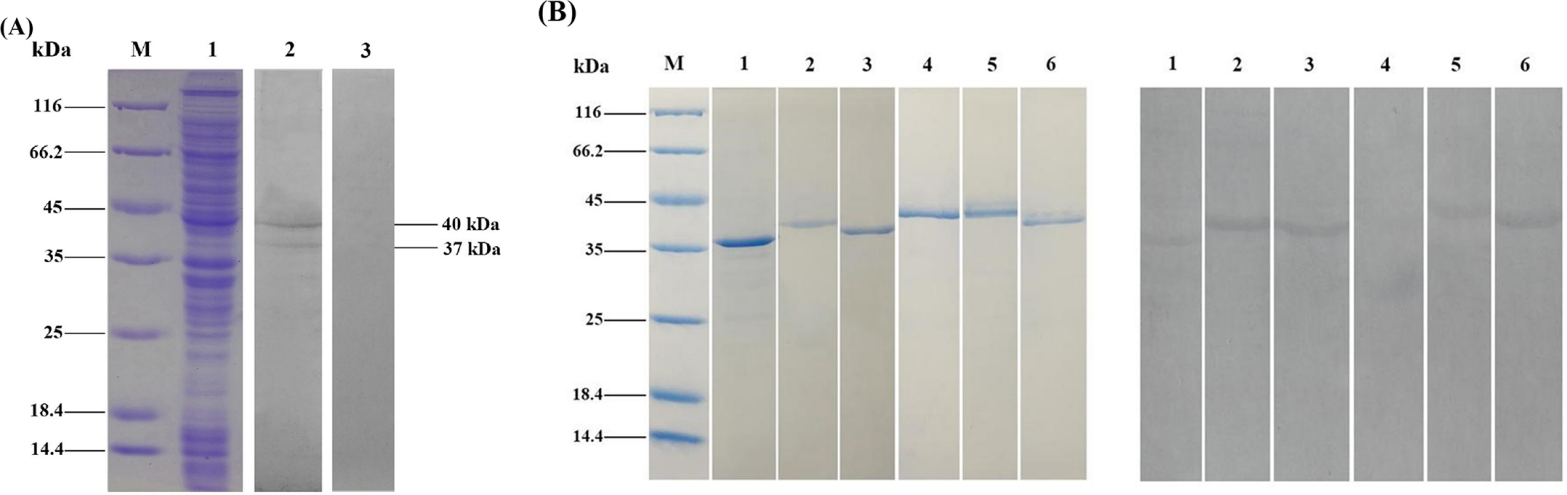
Analysis of the whole-cell lysate proteins of *S*. *iniae* and the immunogenicity of the recombinant proteins by SDS-PAGE and Western-blotting. **(A)** Lane M: molecular mass marker; Lane 1: whole-cell lysate proteins; Lane 2: western blotting analysis using flounder anti-*S*. *iniae* serum; Lane 3: negative control using the healthy flounder serum. **(B)** Lane M: molecular mass marker; Lane 1: rPDHA1; Lane 2: rBMP; Lane 3: rLDH; Lane 4: rOCT, Lane 5: rLOx; Lane 6: rGAPDH.

To screen the immunogenic proteins, the whole-bacterial proteins of *S*. *iniae* was analyzed by western blotting using flounder anti-*S*. *iniae* antibodies, and the results showed that two obvious protein bands with molecular weight (MW) of approximate 37 kDa and 40 kDa were recognized by flounder anti-*S*. *iniae* antibodies, and no band was observed in negative control (Fig 1A). The 37 kDa and 40 kDa protein bands were analyzed by mass spectrometry and six proteins were identified, and the results of theoretical isoelectric point (PI), theoretical MW, mascot score, match peptide number, protein coverage and accession numbers in Genbank of the six proteins were given in Table 2. The 37 kDa immunogenic protein matched 2 peptides with pyruvate dehydrogenase E1 subunit alpha (PDHA1), 1 peptide with BMP family ABC transporter substrate-binding protein (BMP) and 2 peptides with L-lactate dehydrogenase (LDH). While the 40 kDa immunogenic protein matched 4 peptides with ornithine carbamoyltransferase (OCT), 1 peptide with lactate oxidas (LOx) and 5 peptides with glyceraldehyde-3-phosphate dehydrogenase (GAPDH). All of them obtained high mascot scores.

**Table 2:** Mass spectrometric results of the 37 kDa and 40 kDa protein.

Molecular mass	Protein Name	Accession No.	Theoretical pI	Theoretical MW_ _(Da)	Mascot Score /No. of Match Peptide	Protein Coverage (%)
**37kDa**	Pyruvate dehydrogenase E1 subunit alpha	WP_003099247.1	5.00	35608	143/2	20
	BMP family ABC transporter substrate-binding protein	WP_003099521.1	8.3	36551	85/1	11
	L-lactate dehydrogenase	WP_003099652.1	4.79	35364	201/2	8
**40kDa**	Ornithine carbamoyltransferase	WP_003100983.1	4.99	38124	339/4	18
	Lactate oxidase	CAA68903.1	7.36	44207	74/1	13
	Glyceraldehyde-3-phospate dehydrogenase	ACX85247.1	5.16	35962	466/5	23

### Production and immunogenicity of recombinant proteins

The results of SDS-PAGE revealed that the recombinant proteins of PDHA1 (rPDHA1), BMP (rBMP), LDH (rLDH), OCT (rOCT), LOx (rLOx) and GAPDH (rGAPDH) of *S*. *iniae* were successfully expressed in *E*. *coli* BL21 (DE3) after IPTG induction, and the purified recombinant proteins appeared as a single band at 37, 40, 38, 43, 43 and 41 kDa respectively, matching the predicted molecular masses of the six proteins. The results of western blotting showed that the six recombinant proteins, except rOCT, could be recognized by flounder anti-*S*. *iniae* antibodies (Fig 1B). No visible band was found when the anti-*S*. *iniae* antibodies were replaced by healthy flounder serum.

### Immunoprotective effects of six recombinant proteins

**Fig 2:**
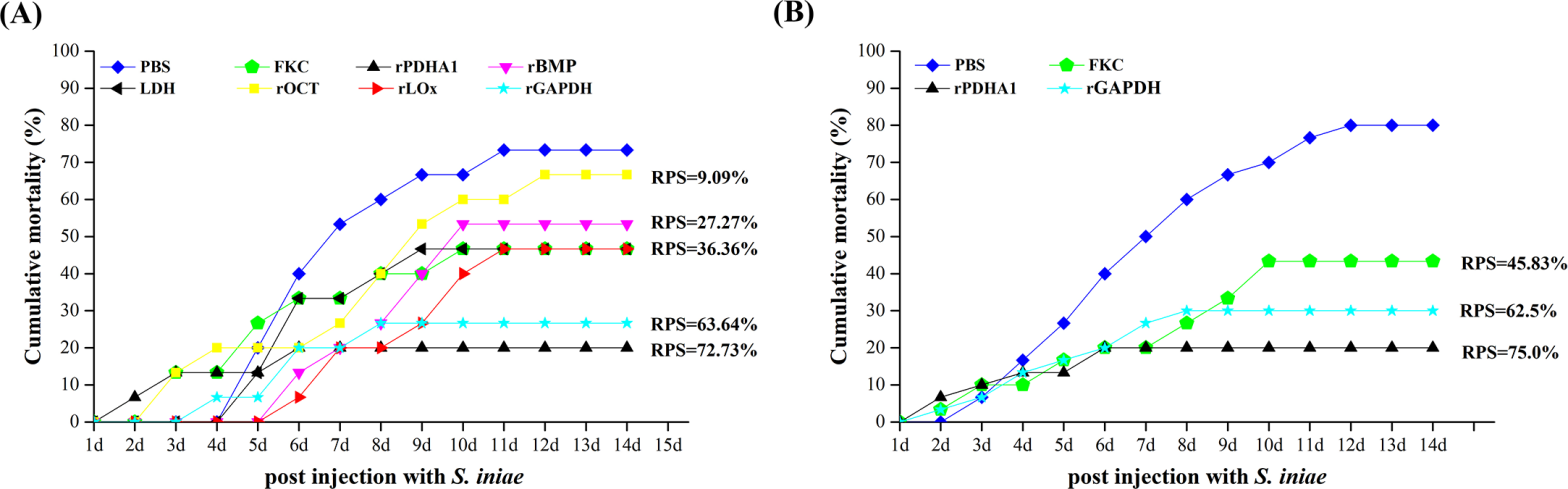
Cumulative mortality of flounder after challenged with live *S*. *iniae*. RPS was calculated with a PBS group as the control.

The flounders were challenged with live *S*. *iniae* at 5 weeks post booster vaccination with the six recombinant proteins, and the results showed that the cumulative mortalities of the control and FKC groups were 73.33% and 46.67%, respectively, which corresponded to a RPS of 36.36% in FKC group. The fish vaccinated with different recombinant proteins had significantly lower cumulative mortality than the control group. The cumulative mortality rates of vaccinated fish were 53.33%, 66.67%, 46.67%, 46.67%, 20% and 26.67% in rBMP, rOCT, rLOx, rLDH, rPDHA1 and rGAPDH groups, corresponding to the calculated RPS of 27.27%, 9.09%, 36.36%, 36.36%, 72.73% and 63.64% respectively (Fig 2A). Notably, in rPDHA1 and rGAPDH groups, which gave a higher RPS than FKC group, the mortality only occurred in the first 7 days and the survival rate of flounder against *S*. *iniae* were significantly increased, suggesting they might be ideal potential vaccine candidates. All the moribund and dead flounder demonstrated typical signs of streptococcicosis including haemorrhage, exophthalmia, abdominal distension, ascites, and lesions of the liver, kidney, spleen, and intestine.

To further investigate the protective efficacy of the selected rPDHA1 and rGAPDH, flounder were immunized with FKC, rPDHA1, rGAPDH or PBS once again, and then challenged with 100μl *S*. *iniae* (1.0×10^7^ cfu/ml) at day 35 post-vaccination. The cumulative mortality rate of the PBS injected group increased rapidly, reaching 80% in week 3. The cumulative mortality rates of vaccinated fish were 43.3%, 20% and 30% in FKC, rPDHA1 and rGAPDH groups, corresponding to the calculated RPS of 45.83%, 75% and 62.5% respectively (Fig 2B).

### Flow cytometric analysis

**Fig 3:**
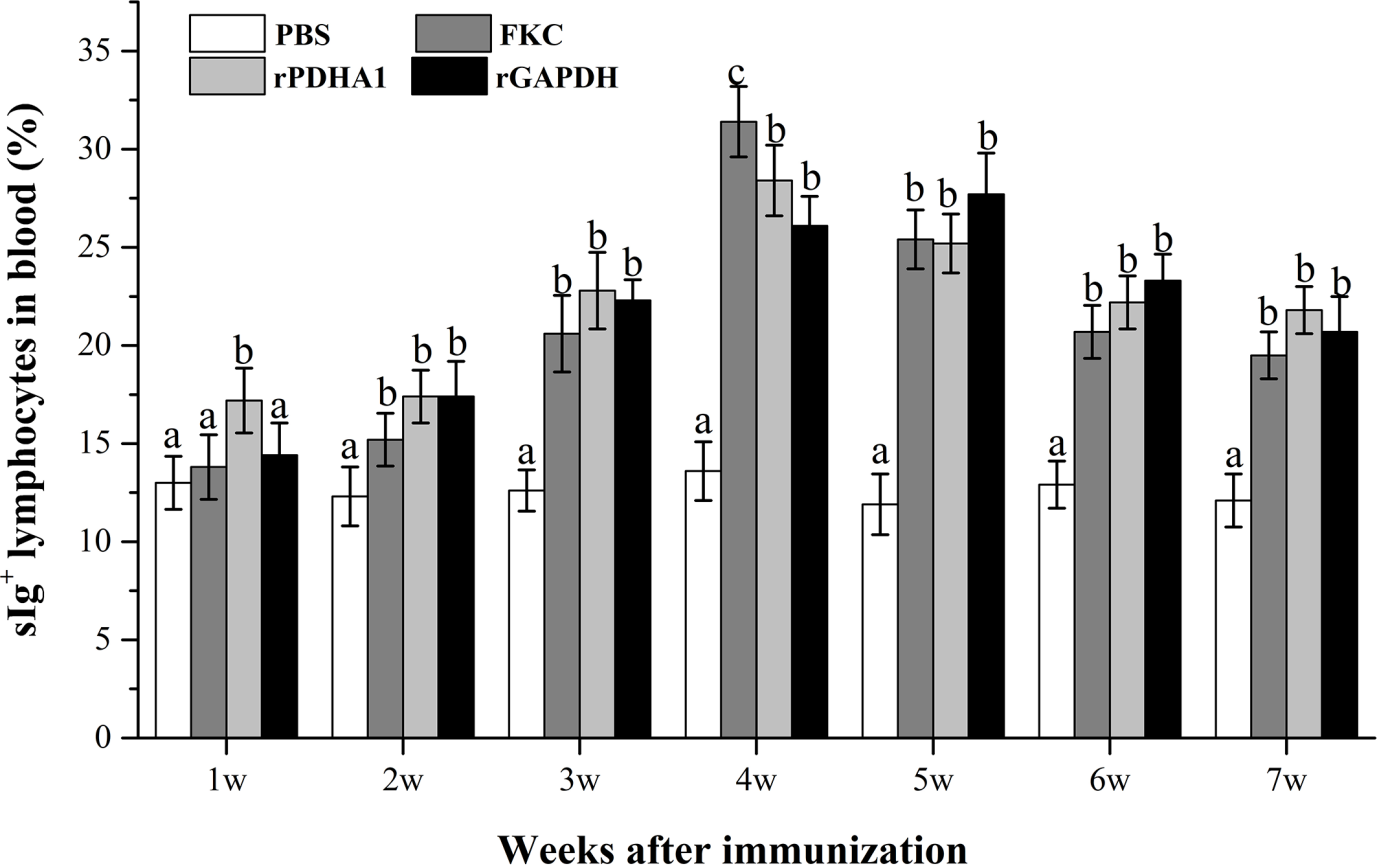
The percentage of sIg^+^ lymphocytes in PBLs of flounder at different time points after immunization. The results are presented as the means ± SEM of six fish. Different letters above the bar represent the statistical significance (*p* < 0.05) compared to each other at same time point.

The PBLs were isolated from the peripheral blood of vaccinated fish, and analyzed for forward scatter (FSC) and sideward scatter (SSC) pattern which represented the cell size and granularity respectively. The dot plots of the gated lymphocytes and the fluoresce histograms of FKC, rPDHA1 and rGAPDH immunized fish at week 4 post vaccination were shown in S1 Fig. The changes in percentages of the sIg^+^ lymphocytes in PBLs of vaccinated fish during 7 weeks were summarized in Fig 3. The percentage of sIg^+^ lymphocytes in PBLs induced by FKC, rPDHA1 and rGAPDH vaccination all showed a variation tendency that increased firstly and then decreased, and still obviously higher than the control at week 7. Post immunization, the percentage of sIg^+^ lymphocytes in PBLs significantly increased from week 1 in rPDHA1 group as compared with the control (*p*<0.05), and peaked at week 4. While in rGAPDH and FKC groups, it demonstrated significant increase from week 2, and reached the peak at week 5 and week 4 respectively. There were no significant differences in the three immunized groups except at week 4 where FKC induced a higher percentage of sIg^+^ lymphocytes in PBLs (31.3%) than rPDHA1 (28.4%) and rGAPDH (26.1%).

### Detection of total IgM in serum

As shown in S2 Fig, the production of total serum IgM presented a tendency to increase first and then decrease gradually in all vaccinated groups. Compared with the control group, the levels of total serum IgM in rGAPDH immunized fish increased from week 1, and kept in the peak value at week 4 and 5 where they were significantly higher than that in rPDHA1 and FKC groups (*p*<0.05), and then went down to a lower level than the other two groups at week 6 even though the difference was not statistically significant (*p*>0.05). In rPDHA1 and FKC immunized fish, the levels of total IgM obviously increased from week 2 and peaked at week 6, and no significant differences were observed between them during 7 weeks (*p*>0.05).

### Detection of specific IgM against *S*. *iniae* in serum

**Fig 4:**
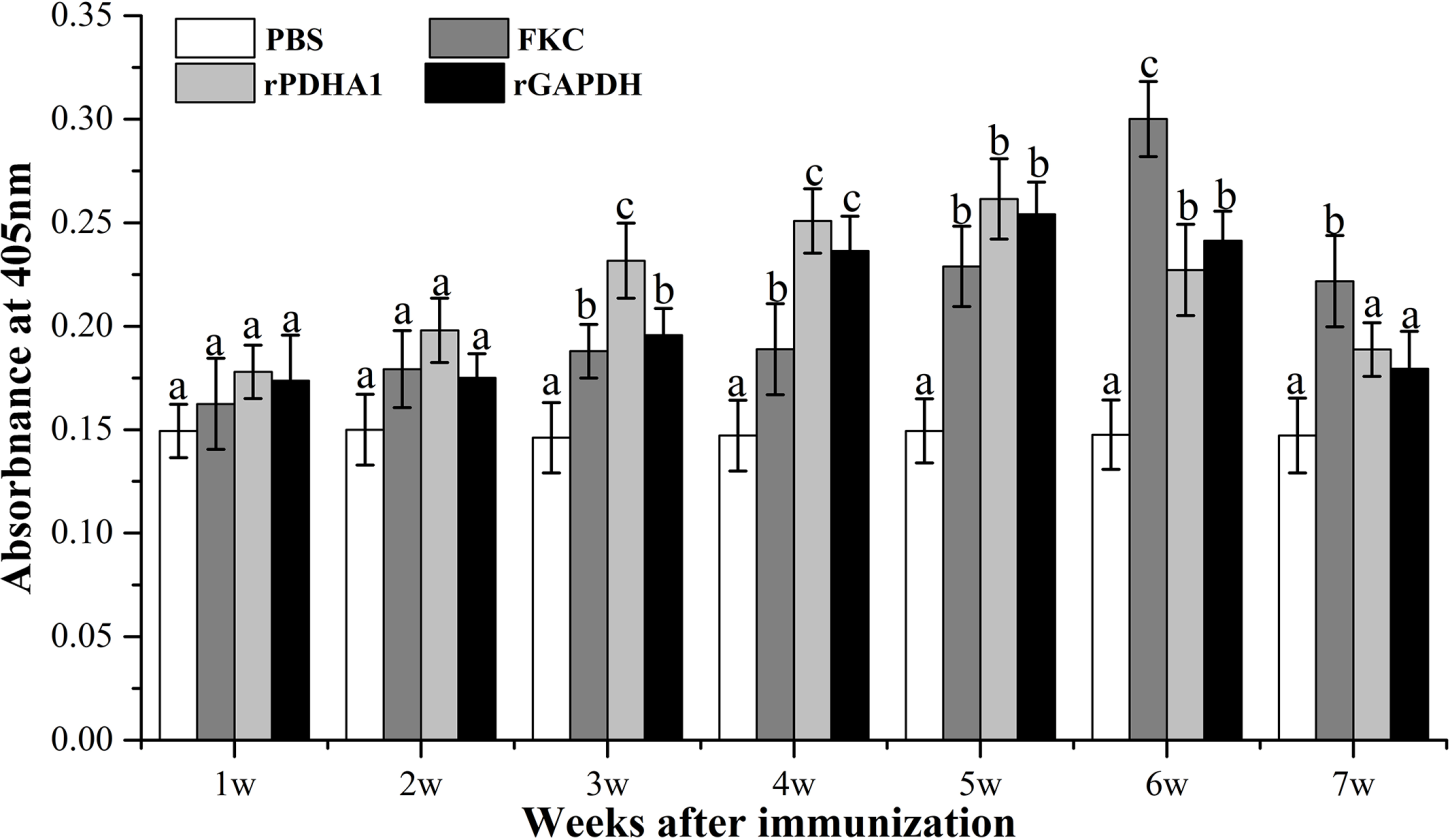
Specific antibodies against *S*. *iniae* in vaccinated flounder from 1 to 7 week. Values are shown as means ± SEM of six fish. Different letters above the bar represent statistical differences (*p* < 0.05).

ELISA analyses showed that the FKC, rPDHA1 and rGAPDH all could induce the production of specific IgM against *S*. *iniae* that exhibited similar variation trend to the total serum IgM (Fig 4). The levels of specific IgM in serum increased dramatically from week 3 in the three vaccinated groups, and arrived at the maximum value at week 5 in rPDHA1 and rGAPDH groups and at week 6 in FKC group, even higher than the control at week 7. The levels of specific IgM in rPDHA1 and rGAPDH groups were higher at week 3, 4 and 5, but lower at week 6 and 7, than those in FKC group. Compared rPDHA1 with rGAPDH groups, though the specific IgM levels in rPDHA1 group were higher, there were no significant differences (*p*>0.05) between them during 7 weeks except at week 3 (*p*<0.05).

## Discussion

Immunogenic proteins have been identified to develop candidate vaccine in several bacteria, including *E*. *tarda*, *V*. *alginolyticus* and *V*. *parahaemolyticus*, using mouse or rabbit anti-bacteria antibody combining with mass spectrometry analysis [36–38]. However, a previous study revealed that some antigenic proteins identified by rabbit polyclonal antiserum failed to react with fish antiserum [30], suggesting that some differences maybe exist in immune responses and antibody isotypes between fish and mammals. In the present study, in order to develop candidate vaccines against *S*. *iniae* infection in flounder and avoid the problem described above, immunogenic proteins of *S*. *iniae* were screened using flounder anti-*S*. *iniae* antibody by western blotting, and two positive protein bands were recognized,. Six recombinant proteins, PDHA1, BMP, LDH, OCT, LOx and GAPDH, with high purity were obtained and analyzed, and it was found that flounder anti-*S*. *iniae* antibodies could react with all recombinant proteins except rOCT, suggesting that these components of *S*. *iniae* could induced flounder to produce antibodies. As for rOCT, it was possibly because of the lower abundance of OCT in *S*. *iniae* that produced too little antibodies in founder. Some low abundant proteins may play key roles in bacterial infection. It has been reported that OCT of outer-surface proteins in bacterial pathogens play an important role in pathogenesis, and rOCT could provide effective protection against *S*. *agalactiae* in tilapia [39], thus more studies about OCT of *S*. *iniae* are required.

References confirm that recombinant immunogenic proteins purified from *E*. *coli* and mixed with suitable adjuvants can provide high levels of protection against *S*. *iniae* [21, 40]. In our work, the immunoprotection of the six recombinant proteins was investigated in flounder model, and the RPS in immunized groups of rPDHA1, rBMP, rLDH, rOCT, rLOx and rGAPDH was 72.73%, 27.27%, 36.36%, 9.09%, 36.36% and 63.64% after live *S*. *iniae* challenge respectively, while the RPS in FKC group was also 36.36%, indicating that they were all protective antigens and could provide different degrees of protection against *S*. *iniae* infection. It has been reported that FKC vaccines with homologous capsular serotype of the challenge strain provide enduring 100% protection [41], which is different from our results, this may be due to the difference in concentrations of FKC for vaccination and live *S*. *iniae* for challenge, or the difference in bacterial strains. Moreover, previous study found that FKC enriched with bacterial extracellular products would provide superior protection against *S*. *iniae* than FKC [42]. Thus, bacterial extracellular products might be one reason why the FKC just provide a 36.36% RPS in this study. The references have reported that the immune responses are associated with RPS, and the higher levels of immune response might produce higher RPS [22, 26]. RPS is an important parameter to evaluate the effects of vaccines. The rPDHA1 and rGAPDH provided higher RPS than other recombinant proteins as well as FKC in this study. To our knowledge, the report about PDHA1 as potential vaccine was limited, and we proved for the first time that the rPDHA1 provided high protection against *S*. *iniae* in flounder. The GAPDH of *S*. *iniae* was identified as immunogenic protein in the present study, which was in line with some studies on Streptococcus spp. [19, 23, 43]. Based on these results, rPDHA1 and rGAPDH were chosen as potential subunit vaccine candidates to further evaluate their protective efficacy in flounder. Furthermore, rLDH and rLOx provided the same RPS as FKC in this study, which might also be developed as candidate vaccine to resist *S*. *iniae* infection. LDH of *E*. *acervulina* can produce protection against *E*. *acervulina* infection and DNA vaccine carrying *E*. *acervulina* LDH antigen gene can induce protective immunity [44, 45]. While the LOx identified in this study has not been previously demonstrated to be immunogenic in other bacteria. Therefore, further researches are needed to clarify their protective effect against *S*. *iniae* infection.

It is reported that intraperitoneal administration of a single *S*. *iniae* vaccine gives superior efficacy in comparison with the immersion and oral routes [9, 42].Previous studies confirme that FCA is the most widely used and most effective adjuvant for experimental purposes, and FCA outcompeted the other adjuvants [40, 46]. In this study, flounder was intraperitoneally injected with rPDHA1 and rGAPDH or FKC equally mixed with FCA, and it was demonstrated that sIg^+^ lymphocytes in PBLs, the levels of total serum IgM and specific IgM were significantly enhanced in immunized flounder, showing a variation trend to increase first and then decrease. The specific IgM levels in rPDHA1 and rGAPDH groups were higher before week 5 and peaked earlier than FKC group; however, no significant differences in total IgM levels of three immunized group except week 4 and 5. The sIg^+^ lymphocytes in PBLs of vaccinated flounder were found that no significant differences presented in the three immunized groups except week 4 where the FKC group was the highest. These results showed that rPDHA1 and rGAPDH could induce a strong humoral immune response similar to FKC, but provide a higher RPS than FKC, which might be due to the abundance of immunogenic proteins in FKC was lower than recombinant proteins PDHA1 and GAPDH. Previous studies found that several surface proteins play important roles in bacterial infection, but their abundance or immunogenicity may be very low, these may be the reasons that some formalin-inactivated vaccines induce low protective effects [47]. There was a difference in RPS between the two challenge tests in FKC group, which might be due to the differences of two batches of the flounder. While rGAPDH and rPDHA1 gave similar high protection against *S*. *iniae* in the two challenge tests, indicating the two proteins as subunit vaccines could provide stable and effective protection against *S*. *iniae*. Our results also suggested that the sIg^+^ and IgM levels do not always correlate with RPS. Similarly, previous studies also indicate that high levels of immune response induced by subunit vaccines may have no direct relation to RPS [27], thus more studies on their relationship are required, which is helpful for developing an appropriate methodology for the evaluation of vaccine efficacy in fish.

## Conclusions

In conclusion, six immunogenic proteins were identified from *S*. *iniae* and their recombinant proteins were obtained, and then rPDHA1 and rGAPDH were chosen as potential vaccine candidates based on their higher RPS than FKC. The protective efficacy of rPDHA1 and rGAPDH were further evaluated, and they were found to induce higher levels of total IgM, specific IgM in serum, proliferation of sIg^+^ lymphocytes in PBLs, and provide high protection against *S*. *iniae*, which indicated that PDHA1 and GAPDH are promising vaccine candidates against *S*. *iniae* infection. These results, together with our previous studies on immunogenic proteins from *E*. *tarda*, *V*. *anguillarum and V*. *ichthyoenteri*, would provide important information for us to develop the effective subunit vaccines, especially the multi-vaccine, against different bacterial pathogens in flounder.

## Supporting information

S1 Checklist NC3Rs ARRIVE guidelines checklist. The ARRIVE guidelines describes the number and specific characteristics of animals used; details of housing and husbandry; and the experimental, statistical, and analytical methods.
(PDF)

S1 Fig Flow cytometric analysis of lymphocytes labelled with anti-serum IgM mAb 2D8. A1, B1 and C1 represent lymphocytes in FKC, rPDHA1 and rGAPDH group gated (R1) on a forward scatter (FSC) versus side scatter (SSC) dot plot, respectively. A2, B2 and C2, vaccinated fish, combined (smoothed) a FITC fluorescence histogram of gated lymphocytes (R1) showing the percentages of sIg^+^ lymphocytes (scale of M) in peripheral blood at week 4 after immunization.
(TIF)

S2 Fig Total serum antibodies in vaccinated flounder from 1 to 7 week. Values areshown as means ± SEM of six fish. Different letters above the bar represent statistical differences (*p* < 0.05).
(TIF)
