# Supplementary figures and images for: Identification of immunogenic proteins and evaluation of recombinant PDHA1 and GAPDH as potential vaccine candidates against Streptococcus iniae infection in flounder (Paralichthys olivaceus)
Source: PLoS One. 2018 May 30;13(5):e0195450. doi: 10.1371/journal.pone.0195450 (PMC5976140; doi:10.1371/journal.pone.0195450)

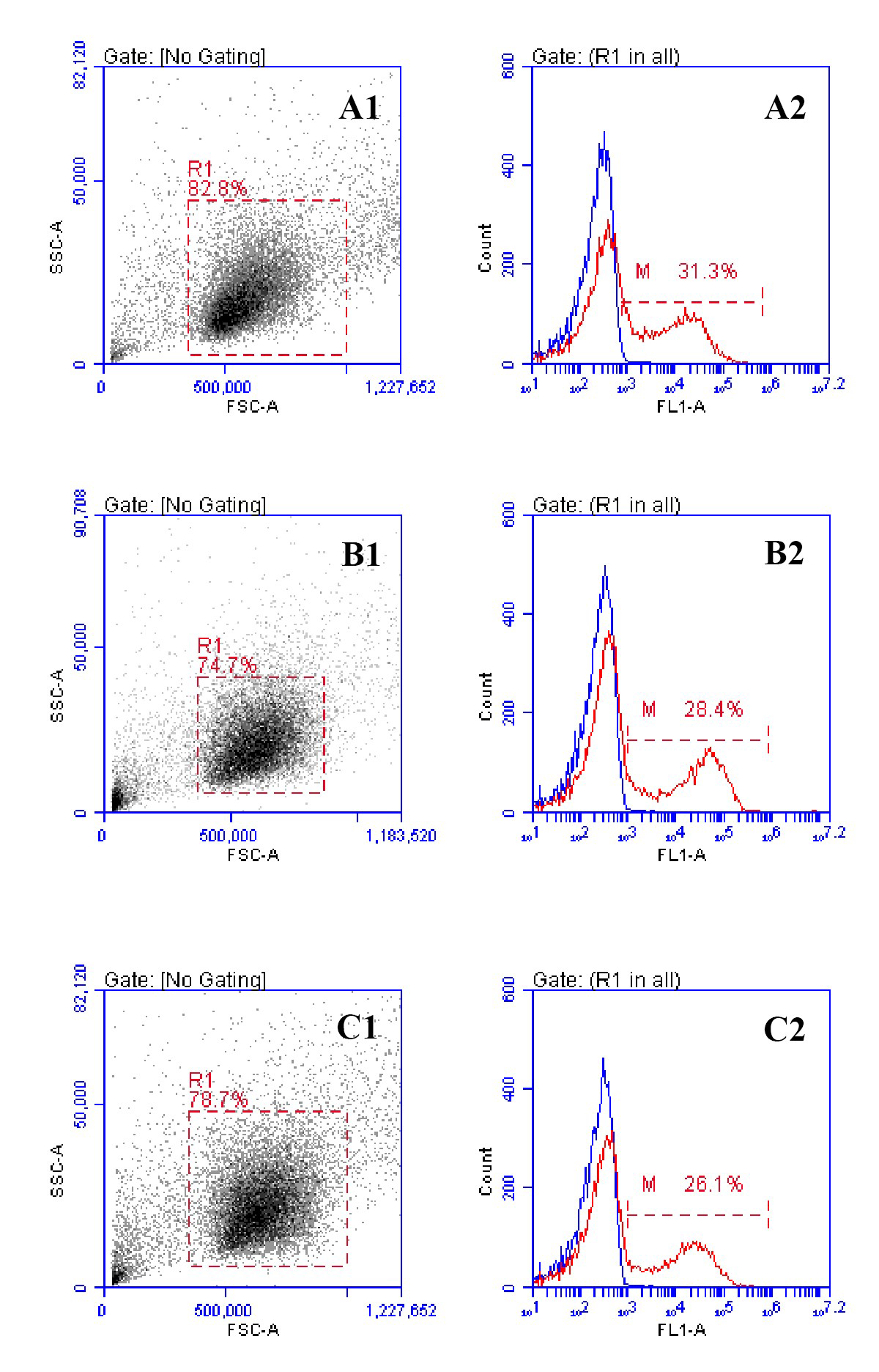

Supplement: S1 Fig — A1, B1 and C1 represent lymphocytes in FKC, rPDHA1 and rGAPDH group gated (R1) on a forward scatter (FSC) versus side scatter (SSC) dot plot, respectively. A2, B2 and C2, vaccinated fish, combined (smoothed) a FITC fluorescence histogram of gated lymphocytes (R1) showing the percentages of sIg+ lymphocytes (scale of M) in peripheral blood at week 4 after immunization. (TIF) [file pone.0195450.s002.tif]

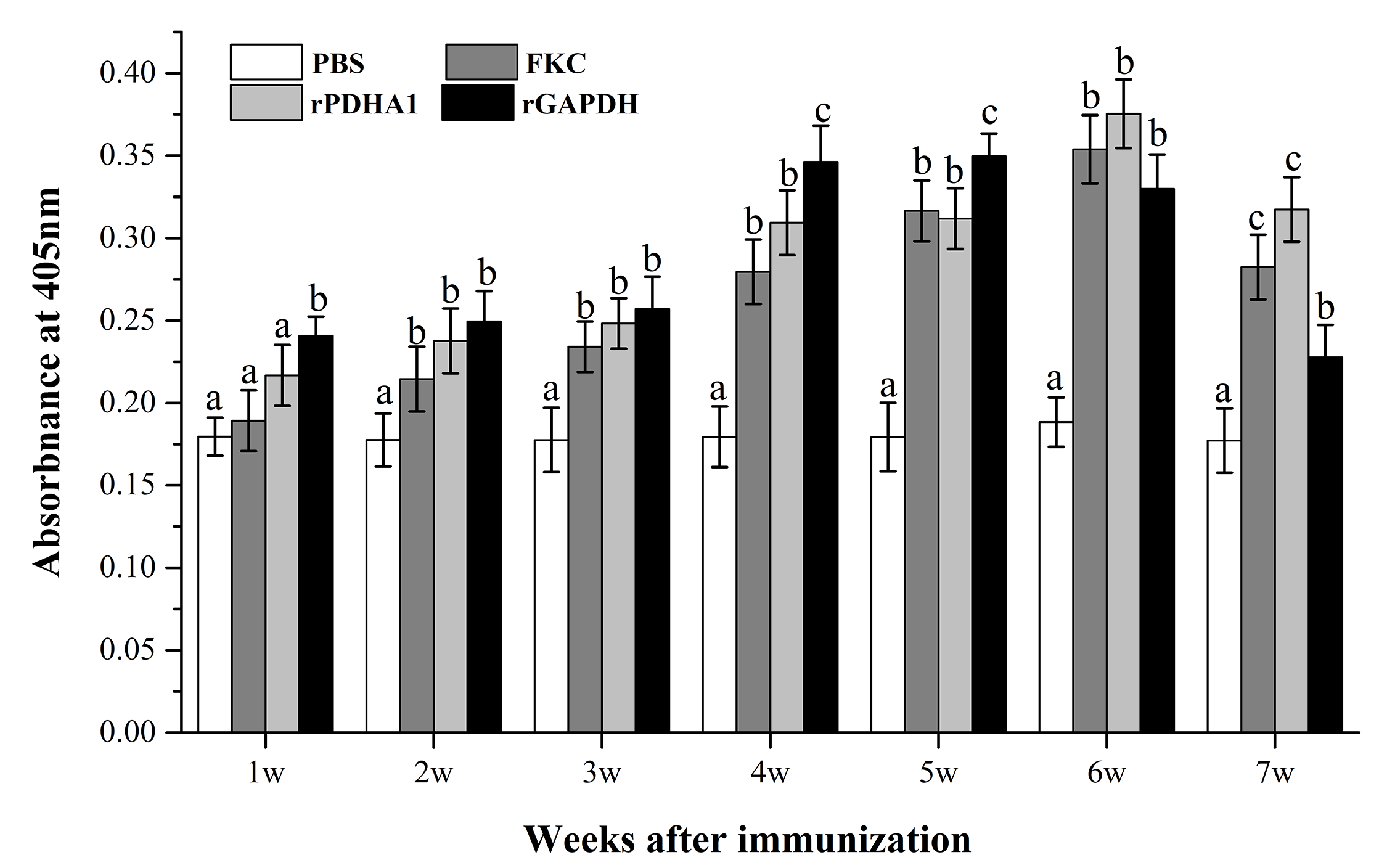

Supplement: S2 Fig — Values areshown as means ± SEM of six fish. Different letters above the bar represent statistical differences (p < 0.05). (TIF) [file pone.0195450.s003.tif]
